# A graphene-based Fabry-Pérot spectrometer in mid-infrared region

**DOI:** 10.1038/srep32616

**Published:** 2016-08-30

**Authors:** Xiaosai Wang, Chen Chen, Liang Pan, Jicheng Wang

**Affiliations:** 1 School of Science, Jiangsu Provincial Research Center of Light Industrial Optoelectronic Engineering and Technology, Jiangnan University, Wuxi 214122, China; 2 School of Mechanical Engineering and Birck Nanotechnology Center, Purdue University, West Lafayette, Indiana 47907, USA; 3 Key Laboratory of Semiconductor Materials Science, Institute of Semiconductors, Chinese Academy of Sciences, 912, Beijing 100083, China

## Abstract

Mid-infrared spectroscopy is of great importance in many areas and its integration with thin-film technology can economically enrich the functionalities of many existing devices. In this paper we propose a graphene-based ultra-compact spectrometer (several micrometers in size) that is compatible with complementary metal-oxide-semiconductor (CMOS) processing. The proposed structure uses a monolayer graphene as a mid-infrared surface waveguide, whose optical response is spatially modulated using electric fields to form a Fabry-Pérot cavity. By varying the voltage acting on the cavity, we can control the transmitted wavelength of the spectrometer at room temperature. This design has potential applications in the graphene-silicon-based optoelectronic devices as it offers new possibilities for developing new ultra-compact spectrometers and low-cost hyperspectral imaging sensors in mid-infrared region.

Compared to simple photodetection, a spectroscopic study which resolves a spectrum of signal as a function of wavelength adds another dimension into detection and reveals much more information of the object, such as its temperature, motion and composition^1^,^2^,^3^,^4^. Spectra in mid-infrared are particularly desirable in various fields such as environmental monitoring, chemical sensing and astronomical detecting, because fingerprints of many materials fall in this spectral region^5^,^6^,^7^,^8^,^9^,^10^,^11^,^12^,^13^,^14^,^15^,^16^. A typical infrared spectrometer consists of a filter and a detector. The filter extracts a monochromatic beam from a broadband source and directs it to a subsequent detector. By scanning this monochromatic wavelength, the reflection or transmission spectroscopy of the object can be captured. Gratings are commonly used in spectrometers as dispersive filters to separate the incident light into different directions based on their wavelengths. Instead of using gratings, Fabry-Pérot spectrometers use optical cavities consisting of two parallel highly reflecting mirrors as filters and select a particular wavelength by shifting the relative optical phase of these two mirrors. Fabry-Pérot spectrometers can achieve excellent resolving power and operates with non-point-like light sources^17^. However, typical Fabry-Pérot spectrometers have large footprints and require sophisticated scanning systems. Creating ultra-compact Fabry-Pérot spectrometers that are compatible with complementary metal-oxide-semiconductor (CMOS) technology will allow convenient access to spectroscopy information and also can open up the possibility of performing fast and sensitive hyperspectral imaging in mid-infrared region^18^,^19^,^20^.

In this paper we report an ultra-compact Fabry-Pérot spectrometer design based on graphene plasmonics and show the response of this newly proposed structure through theoretical and numerical studies. This Fabry-Pérot spectrometer couples propagating beams into a monolayer graphene as surface waves and uses a pair of Bragg reflectors to form an optical cavity of hundreds of nanometers in size. A scanning gate voltage regulates the optical phase shift between the Bragg reflectors to select the transmission wavelength by electrically controlling the optical property of the graphene layer. A high-transmission peak presents when constructive interference occurs between transmitted beams. The proposed structure is ultra-compact in size (about several micrometers) and is compatible with those graphene-based photodetectors^8^,^9^,^10^,^11^,^21^. We expect this graphene-based spectrometer can make more versatile measurement compared to traditional ones and thus benefit the research areas where mid-infrared spectroscopy is of great importance.

## Results

Detection of mid-infrared light has attracted many research interests since the first observation of infrared radiation from sunlight in the early 1800 s^14^. And the discovery of variable bandgap semiconductor *Hg*_1−*x*_*Cd*_*x*_*Te* in the 1950 s has provided unprecedented degree of freedom to design infrared detectors^22^. By tuning the composition of *Cd x*, the bandgap of *Hg*_1−*x*_*Cd*_*x*_*Te* increases from a negative value to a positive one, allowing the detection region to cover the whole mid-infrared spectral range. More recently, mid-infrared photodetectors have been made using quantum-well and quantum-dot structures on III-V materials^10^,^11^,^23^, where the photoelectrons are excited from bound states that result from quantum confinement. However, wide applications of these above-mentioned mid-infrared detectors are hindered by the complexities in device fabrication and the requirements of cryogenic cooling to achieve high sensitivity^10^,^11^,^21^,^23^.

Graphene, a monolayer of carbon atoms arranged in a honeycomb lattice, offers a promising platform to overcome these obstacles^8^,^9^,^10^,^11^,^12^,^21^. First, the graphene-based techniques can benefit from the mass production of graphene film. Second, graphene interacts strongly with light^11^,^21^,^24^,^25^, which gives sufficient signal even at room temperature. The strong graphene-light interaction also leads to the small footprint of graphene-based photodetectors since the resultant large effective refractive index can greatly shrink the wavelength of light. In addition, graphene-based photodetectors outperform the traditional detectors in many other aspects^8^,^9^,^10^,^11^,^21^,^24^, for example, graphene-based photodetectors have wide detection range, covering all the telecommunication bandwidth as well as mid- and far- infrared, because of the unique linear bandstructure of Dirac fermions in graphene; graphene-based detectors also have high operation speed due to the high carrier mobility of graphene, exceeding 200,000 *cm*^2^ *V*^−1^ *s*^−1^ at room temperature, which is among the highest in known materials; graphene-based techniques are compatible with CMOS processing because of graphene’s two dimensional character and its great dynamic tunability, which enables cost-effective integration of electronics and optics onto a single chip; graphene-based detectors also have a low dark current in general and thus low energy consumption, making them an ideal candidate in high-sensitivity and low-noise detection systems. The unique electronic structure and dynamics of graphene can also support prorogation of surface plasmon polaritons (SPPs), which are polariton mode of photon and electron density waves at a conductor and dielectric interface. Propagating light can be efficiently coupled into SPPs, which have many scientific and engineering applications in computing, data storage, communication, nanoelectronic and nanophotonics^26^,^27^,^28^,^29^,^30^,^31^,^32^,^33^,^34^.

**Figure 1:**
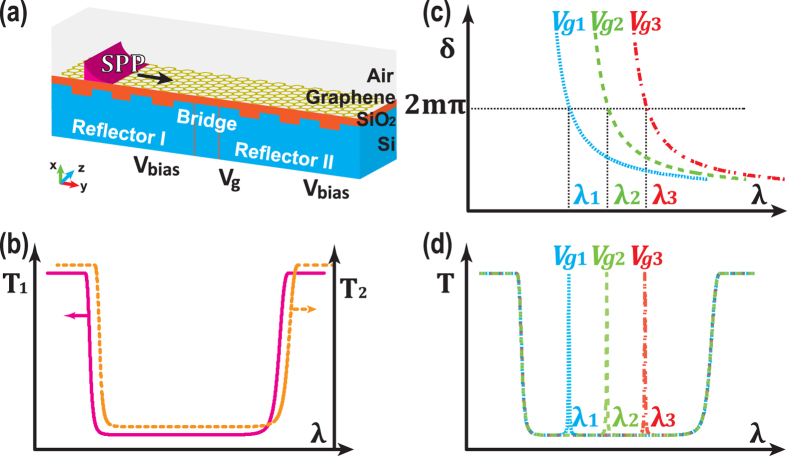
Schematic of the structure of a graphene-based Fabry-Pérot spectrometer and its working principle. (**a**) Schematic of the graphene-based Fabry-Pérot spectrometer. Surface plasmon polaritons (SPPs) in graphene propagate across two Bragg reflectors and a bridge between them that are modulated by different voltages. The direction of the propagation of SPPs is indicated by the arrow on top of the graphene layer. (**b**) Transmission spectrum *T*_1_ (*T*_2_) if only Bragg Reflector I (II) is present. (**c**) Optical phase shift *δ* between two reflectors as a function of wavelength *λ*. *δ* is inversely proportional to *λ*. It reaches 2*mπ* at different wavelengths when different gate voltages are applied. (**d**) Overall transmission spectra of the whole structure for different gate voltages. Transmission peak is at the wavelength where *δ* equals to 2*mπ*.

The schematic and working principle of our proposed spectrometer are illustrated in Fig. 1. The structure is composed of two Bragg reflectors, achieved by periodically modulating the surface conductivity of graphene using a silicon based grating structure, and a bridge between these two reflectors. During its operation, the broadband mid-infrared irradiations will be first coupled into SPPs using surface gratings^29^,^30^,^35^,^36^. The excited SPPs will then propagate along the graphene layer towards the pair Bragg reflectors. Because of the electrically-controlled phase shift between the Bragg reflectors, only a narrowband of spectrum can pass through and reach the receiving detector and the rest will be reflected away. Therefore, a spectrum of the incident mid-infrared light can be captured by scanning the transmission peak of the Bragg reflector pair. As shown in Fig. 1(a), the two Bragg reflectors are subjected to a biased voltage *V*_*bias*_ while the bridge is subjected to a gate voltage *V*_*g*_. The two voltages are electrically isolated using a thin layer of silica (*SiO*_2_). When *V*_*bias*_ is applied between graphene (*Graphene*) and highly-doped silicon (*Si*) spaced by *SiO*_2_, the induced electric field has a periodic modulation due to the grating structure. Since the thickness of *SiO*_2_ *t* is typically small in our model compared to the width of trench in the silicon grating, we negelect the edge effect and assume the field is given by the solution in large parallel plate capacitor 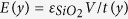, where *t*(*y*) indicates thickness is a periodic step function of *y* and 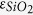 is the relative permittivity of *SiO*_2_ layer. Thus, the surface charge density of graphene can be approximated using Gauss’s law 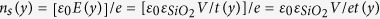, where *ε*_0_ is the vacuum permittivity and *e* is the elementary charge. The change of surface charge density modifies the electron population in graphene, leading to the modulation of Fermi level *E*_*F*_ according to 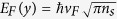, where *v*_*F*_ is the Fermi velocity of electron, 10^6^ *m*/*s* in graphene. The change in Fermi level in turn affects the scattering of electrons, characterized by the relaxation time 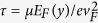. Here *μ* is the carrier mobility in graphene. In the mid-infrared spectral region, the optical properties of a monolayer graphene can be characterized by a complex surface conductivity *σ*_*G*_. *σ*_*G*_ is generally attributed to intraband transitions and interband transitions, which correspond to the first term and the second term of the Kubo formula^37^,^38^, respectively


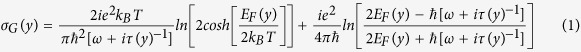


where *k*_*B*_ is the Boltzmann constant, *T* is the temperature, ħ is the reduced Planck constant and *ω* is the angular frequency of optical excitation. Note here we have taken the approximation ħ*ω* ≫ *k*_*B*_*T* and *E*_*F*_ ≫ *k*_*B*_*T*^39^, which is valid through our following discussion, in order to get the form of the second term in Eq. 1. The periodic variation of *σ*_*G*_ results in a periodic variation in the effective refractive index *n*_*eff*_ felt by the propagating SPPs in graphene (see Methods Section for more information). Each boundary causes a partial reflection of the incoming waves due to the mismatch between adjacent effective refractive indices. When the reflected beams add up constructively, high reflection occurs. This happens to a range of wavelengths, known as photonic “stopband”, which is a generic feature when wave propagates in periodically modulating media, as shown in Fig. 1(b). Note here the transmission spectra for Reflector I and Reflector II are the same and the small shift between them is artificially added to aid visualization. So the two Bragg reflectors act as highly reflecting mirrors in a Fabry-Pérot interferometer. Considering the optical phase shift *δ* introduced by the intermediate bridge, the overall reflection and transmission coefficients for the whole structure are given by Fresnel equations in a three layer system


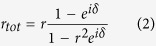



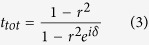


where *r* is the reflection coefficient defined as the ratio between the incident electric field and the reflected electric field when only one Bragg reflector is present. To get the forms in Eqs 2 and 3, we have used the assumption that the media before Reflector I and after Reflector II are the same. The phase shift *δ* = 2*k*_0_*n*_*eff*_ *L* = 4*πn*_*eff*_ *L*/*λ*, where *k*_0_ is the amplitude of wave vector of wavelength *λ* in free space, *n*_*eff*_ is the effective refractive index defined as the real part of the ratio between the wave vector in graphene and *k*_0_, *L* is the physical length of the bridge and the factor 2 comes from the fact that the beam is reflected twice before getting out from the same boundary. As *δ* → 2*mπ* (*m* = 0, 1, 2, …), *r*_*tot*_ → 0 and *t*_*tot*_ → 1 regardless the value of *r*, implying total transmission *T* ≡ |*t*_*tot*_|^2^ = 1, as indicated in Fig. 1(c,d). So the wavelength of transmission peak is given by


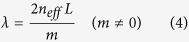


The selective transmission of light can be resolved across the stopband, which essentially works a spectrometer^17^. This analogy between two graphene-based Bragg gratings and Fabry-Pérot interferometer offers a new perspective on the propagation of SPPs in graphene. It provides an intuitive framework to understand and analyze reflection and transmission of propagating waves in the strcture. It is further verified using wave impedance analysis (see Methods Section, which also includes the case *m* = 0). By taking two Bragg reflectors as highly reflecting mirrors and writing *δ* as a pure real number, we have neglected the material losses along propagation in order to emphasize the idea.

The proposed idea is investigated using a commercial software package implementing finite element method–COMSOL Multiphysics. A nominal thickness of graphene is needed to simulate its optical response. This is because the general harmonic response of graphene at the macroscopic scale is described by Maxwell’s equations


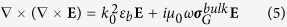


where *ε*_*b*_ is the ion background contribution into the relative permittivity of graphene and 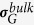 is the bulk conductivity considering that graphene has a finite thickness of *t*_*G*_. By comparing the surface current density ***α*** = *σ*_*G*_***E*** and the bulk current density 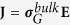, in together with 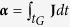, we can approximate 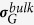 as *σ*_*G*_/*t*_*G*_. Define


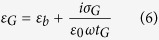


so Eq. 5 can be written as 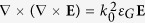, which is the governing equation used in radio frequency (RF) module of COMSOL when relative permittivities of materials are specified. *ε*_*b*_ and *t*_*G*_ are typically chosen to be 2.5 and 1 nm, respectively^25^,^40^,^41^,^42^. Except for graphene, the optical properties of other involved materials, which are characterized by corresponding relative permittivities, are assumed to be constants in the spectral region of interest (from 5 *μm* to 8 *μm*), *i*.*e*. *ε*_*Air*_ = 1, 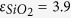 and *ε*_*Si*_ = 11.7. Like many others^25^,^42^,^43^,^44^, we study the properties of graphene with low material losses by only taking the real part of its permittivity. Its losses will be discussed in Discussion Section. The wavelength upperbound of this study is chosen to be 8 *μm* because light absorption of *SiO*_2_ is strong when the wavelength exceeds 8 *μm*^45^.

**Figure 2:**
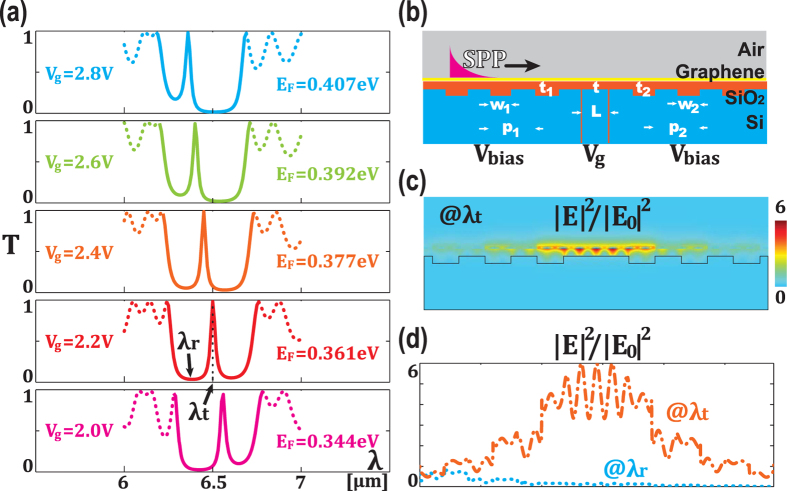
Typical performance of the proposed Fabry-Pérot spectrometer. (**a**) Overall transmission spectrum of the whole structure as a function of gate voltage *V*_*g*_. (**b**) Cross-section view of the proposed structure: *t* is the thickness of silica layer; *L* is the length of bridge; *t*_1_ (*t*_2_), *w*_1_ (*w*_2_) and *p*_1_ (*p*_2_) are the depth of the trench, width of the trench and period of the grating in Reflector I (II), respectively. (**c**) 2D distribution of |**E**|^2^ normalized by |**E**_0_|^2^ at excitation wavelength *λ*_*t*_ indicated in panel (a), where |**E**_0_|^2^ is the value at the leftmost edge of the structure. Outline of silicon grating is overlaid on top for visual aids. (**d**) Comparison between profiles of |**E**|^2^/|**E**_0_|^2^ at *λ*_*t*_ and *λ*_*r*_, along a cutline in *SiO*_2_ right underneath graphene (*Graphene*).

In order to calculate the transmission of the structure, we use a same boundary mode to excite SPPs in graphene for different wavelengths. This is achieved by inserting a graphene waveguide embedded in air into the beam path before the beam passes through the interested structure. The advantage of doing this is twofold: (1) COMSOL can accurately find the boundary mode near the given effective refractive index so the model is robust when changing excitation wavelength as well as geometrical parameters; (2) the single mode excitation gives good definition of transmission that makes the comparison between different wavelengths meaningful. An important parameter in the simulation is the thickness of *SiO*_2_ *t*, as shown in Fig. 2(b). The chosen value of *t* is critical for two reasons: on one hand, *t* should be small compared to *w*_1_ and *w*_2_ to give sufficient spatial modulation of applied electric field; on the other hand, *t* should be large to avoid electric breakdown of *SiO*_2_. The lengths of the bridge and the trenches are also important in order to achieve noticeable shift in spectrum by moderate voltage variations. So the values of voltages (including *V*_*bias*_ and *V*_*g*_), widths (including width of trench in Reflector I *w*_1_ and width of trench in Reflector II *w*_2_), periods (including period of grating in Reflector I *p*_1_ and period of grating in Reflector II *p*_2_) and thicknesses (including *t*, depth of trench in Reflector I *t*_1_ and depth of trench in Reflector I *t*_2_) are dependent on each other and should be optimized globally.

Figure 2(a) shows the transmission spectrum of the structure varies with gate voltage *V*_*g*_ as other parameters are set to be constants *V*_*bias*_ = 3.2 *V*, *w*_1_ = *w*_2_ = 40 *nm*, *L* = 20 *nm*, *p*_1_ = *p*_2_ = 80 *nm*, *t*_1_ = *t*_2_ = 10 *nm* and *t* = 5 *nm*. The numbers of periods in two reflectors are both chosen to be 3. In this case, the distribution of applied electric field is verified to be close to a periodic step function. As expected, a transmission line is present within a stopband formed by two identical Bragg reflectors. The sidelobes outside the stopband drawn as dotted lines are common in Bragg reflectors, resulting from incomplete constructive interference of transmitted beams. Another prominent feature is that the transmission peak gets red shifted as the gate voltage decreases. This is because effective refractive index *n*_*eff*_ increases as *V*_*g*_ decreases (see Methods Section). Recalling from Eq. 4, the transmitted wavelength is proportional to *n*_*eff*_, so the transmission peak is supposed to have a red-shift. To further confirm that the working principle of this spectrometer is as proposed, we plot the distribution of the normalized amplitude square of electric field in Fig. 2(c) at a particular wavelength, *λ*_*t*_, as indicated in the fourth panel of Fig. 2(a). The subscripts “t” and “r” here indicate “transmitted” and “reflected”, respectively. When excitation wavelength equals to *λ*_*t*_, the phase shift introduced by the bridge part is 2*m*_0_*π*, where *m*_0_ is a certain integer, the transmitted beams interfere constructively, leading to complete passing-through of the incident beam. And the electric field in the bridge region, *i*.*e*., the area between two highly reflective mirrors, gets greatly enhanced, as shown by the normalized quantity |**E**|^2^/|**E**_0_|^2^ in Fig. 2(c). |**E**_0_|^2^ is the value taken at the leftmost edge of the plotted structure. Note the outline of silicon grating structure in thin solid line in Fig. 2(c) is overlaid on top of filed distribution to aid visualization. On the contrary, when the excitation wavelength is at *λ*_*r*_, also indicated in Fig. 2(a), the phase shift cannot satisfy the condition of constructive interference between transmitted beams, most of the energy gets reflected back. For comparison, the profiles of |**E**|^2^/|**E**_0_|^2^ along a cutline for two excitation wavelengths *λ*_*t*_ and *λ*_*r*_ are shown in Fig. 2(d). The cutline is drawn right underneath graphene in the *SiO*_2_ side. The normalized value |**E**|^2^/|**E**_0_|^2^ in the bridge area at *λ*_*t*_ is roughly 55 times stronger than that at *λ*_*r*_. The electric field inside the bridge region has multiple peaks, this is because *L* is several times larger than the wavelength of progagating SPPs. In principle, this value can be smaller. So the several micrometer long structure of Fabry-Pérot spectrometer can be further shrinked, making it even more compact in the realm of mid-infrared devices. In both cases in Fig. 2(d), the electric field that is far away from the graphene layer is small, indicating that the scattering of light by the abrupt edges in the structure is not strong.

**Figure 3:**
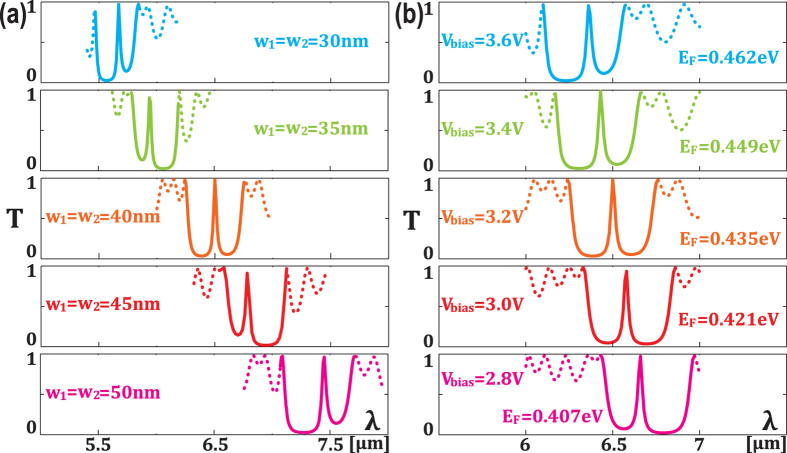
Control of the position of stopband by changing geometric parameters and biased voltage. (**a**) Overall spectrum of the whole structure as a function of widths of trenches in two reflectors *w*_1_ and *w*_2_, with *V*_*g*_ = 2.2 *V* and *V*_*bias*_ = 3.2 *V*. (**b**) Overall spectrum of the whole structure as a function of bias voltage *V*_*bias*_, with *V*_*g*_ = 2.2 *V* and *w*_1_ = *w*_2_ = 40 *nm*.

Another advantage of the proposed structure is its multiple degrees of freedom to engineer the propagation stopband as well as the transmission line. This can be achieved by two ways: one is to change the geometry of the structure and another is to modify the effective rafractive index of graphene, as will be discussed in more details in wave impendance analysis in Methods. Figure 3(a) shows that the stopband can be tuned from around 5.5 *μm* to around 7.5 *μm* only by changing the widths of trenches in two Bragg reflectors. As shown in Fig. 3(b), the biased voltage *V*_*bias*_ offers another knob to dynamically tune the position of stopband. Similar to the trend observed in Fig. 2(a), the decreased *V*_*bias*_ gives rise to the red-shift of stopband. This can be understood by the same reasoning: the reduced voltage leads to increased effective refractive index, which in turn requires longer wavelength to meet the condition of Bragg reflection.

## Discussion

We have demonstrated a new concept of Fabry-Pérot spectrometer based on ideal graphene with low losses. By dynamically tuning the gate voltage applied to the bridge area of graphene, the wavelength of the transmitted line can be continuously changed. More tunability of the structure can be achieved by adjusting the geometric parameters and by dynamically changing the biased voltage applied to graphene. Furthermore, the proposed structure inherits many advantages of graphene-based devices: small footprint, fast speed, broad bandwidth and compatibility with CMOS technologies.

**Figure 4:**
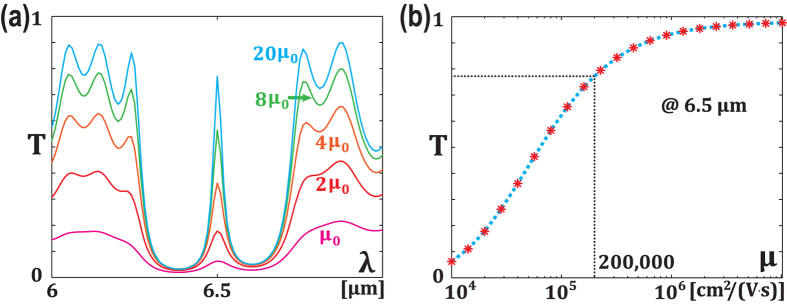
Characterization of material losses in the structure. (**a**) Overall spectrum of the whole structure as a function of carrier mobility (*μ*_0_=10,000 *cm*^2^ *V*^−1^ *s*^−1^). (**b**) Transmission peak at 6.5 *μm* as a function of carrier mobility, with *V*_*g*_ = 2.2 *V*, *V*_*bias*_ = 3.2 *V* and *w*_1_ = *w*_2_ = 40 *nm*.

When material losses are considered, as shown in Fig. 4(a), a transmission peak of quality factor higher than 50 can be achieved when carrier mobility is larger than 20,000 *cm*^2^ *V*^−1^ *s*^−1^ which is a moderate value for graphene on *SiO*_2_^44^. For a mobility of 200,000 *cm*^2^*V*^−1^ *s*^−1^, which is about the best value reported in a suspended graphene monolayer^46^ as indicated by dotted lines in Fig. 4(b), the transmission peak is near 80%. Also, the growth in transmission peak value is small as we artificially increase the carrier mobility. Moreover, many other ways have been proposed to increase carrier mobility of graphene. For example, a relative high value of 60,000 *cm*^2^*V*^−1^ *s*^−1^ have been reported by placing graphene on hexagonal boron nitride^47^. At a mid-infrared wavelength, optical losses can be suppressed using proper doping^42^,^43^. And gain media could be added around graphene to compensate material losses^44^.

We expect the proposed idea of graphene-based Fabry-Pérot spectrometer can be generalized to guide the design of structures using other materials. One example is to push the upperbound of the operation bandwidth into an even longer wavelength by replacing the limiting material *SiO*_2_ with other low loss materials^48^. Also, other dynamically tunable materials rather than graphene^49^,^50^,^51^ can be used to meet certain requirements in desired applications.

## Methods

### Propagation of SPPs in graphene

The analytical solution of SPPs in simple cases can provide us good insights into their properties and help us to understand their response in complex structures. By neglecting the variations in thickness of *SiO*_2_ we can approximate the structure as a multilayer model, where a thin layer of *SiO*_2_ of thickness 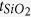 is sandwiched between semi-infinite large air and silicon and a monolayer graphene is next to air and *SiO*_2_. In this waveguide model, graphene is modeled as an infinitely thin surface layer characterized by its complex conductivity *σ*_*G*_, whose imaginary part is typically a positive value in the mid-infrared spectral region of our interest, taking the form *σ*_*G*_ = *σ*_*G*,*r*_ + *iσ*_*G*,*i*_ with *σ*_*G*,*i*_ > 0. In COMSOL, the definition of permittivity is in a different form *σ*_*G*_ = *σ*_*G*,*r*_ − *iσ*_*G*,*i*_, where *σ*_*G*,*i*_ > 0. In this paper, we use the first sign convention unless otherwise stated. One consequence of this property is that the graphene layer can support transverse magnetic (TM) modes in the *Air* − *Graphene* − *SiO*_2_ − *Si* multilayer structure^41^,^52^. The nontrivial components of TM electromagnetic (EM) waves are [*H*_*y*_; *E*_*z*_, *E*_*x*_] if we set the propagation direction of the surface wave to be *y* direction and plane normal direction along *x* axis, as indicated in Fig. 1. Given the graphene plane is *x* = 0 plane, the field components have the form


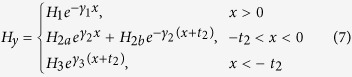


where *γ*_*i*_ (*i* = 1, 2, 3) is defined as 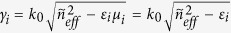 assuming the materials are non-magnetic (*μ*_*i*_ = 1). Here, we have assigned the subscripts *Air*, *SiO*_2_, *Si* as 1, 2, 3, respectively. 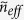 is the effective refractive index of the graphene waveguide, which is dependent on the whole structure. And *ε*_*i*_ and *μ*_*i*_ are relative permittivity and relative permeability of the corresponding material, respectively. In source-free time-harmonic EM fields, electric field **E** is related to magnetic field by


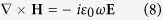


where we have assumed the time-harmonic term takes the form *e*^−*iωt*^. Thus,


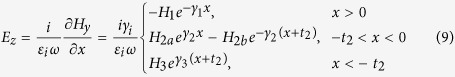


To solve the dispersion relationship of propagating surface waves along graphene, we need the boundary conditions at both *x* = 0 and *x* = −*t*_2_, leading to


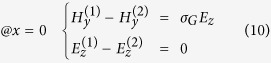



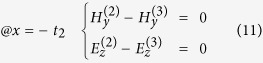


In deriving Eq. 10, we have used the relation between surface current density *α*_*z*_ and local electric field *α*_*z*_ = *σ*_*G*_*E*_*z*_. Substituting Eq. 7 and Eq. 9 into Eqs 10 and 11 and simple rearrangement lead to


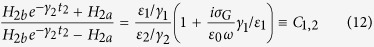



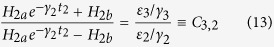


Combining Eq. 12 and Eq. 13 to eliminate *H*_2*a*_ and *H*_2*b*_ gives


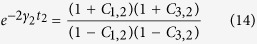


**Figure 5:**
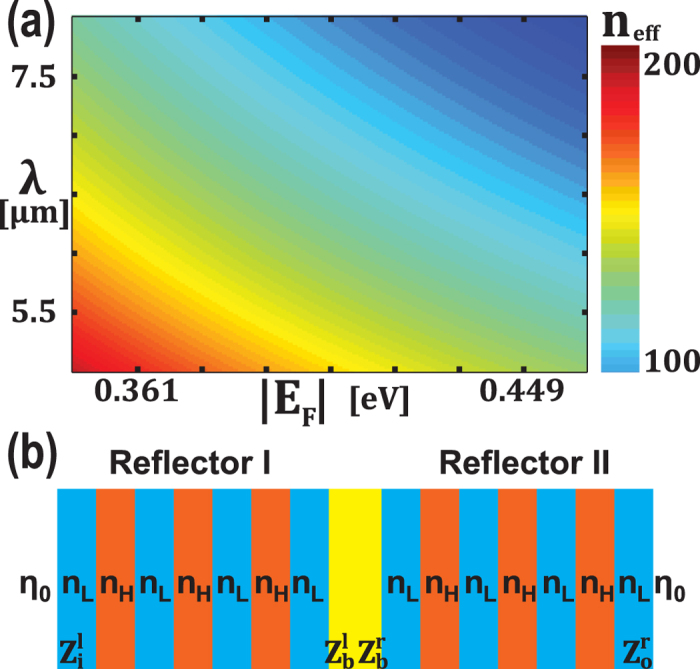
(**a**) Real part of effective reflective index of graphene *n*_*eff*_ as a function of Fermi level *E_F_* and wavelength *λ*. (**b**) Schematic of the proposed structure for wave impedance analysis.

Equation 14 is the general dispersion relation for graphene waveguide in multilayer structures and can be used to calculate effective refractive index 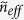. Figure 5(a) shows the real part of 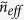 as a function of the strength of Fermi level and the excitation wavelength. When the applied voltage decreases, the Fermi level decreases given a fixed thickness of *SiO*_2_, leading to the increase in *n*_*eff*_. Note when *t*_2_ → ∞, *C*_1,2_ → −1, which is equivalent to^53^


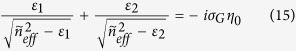


where *η*_0_ is the intrinsic impedance of free space. If *ε*_1_ = *ε*_2_ = *ε*, Eq. 15 reduces to


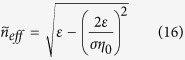


The numerical simulations using the proposed structures are conducted using a commercial finite element method software package, COMSOL Multiphysics. To ensure the same excitation for SPPs in graphene, we set up port boundary conditions for the left and right boundaries. To be specific, we utilize the numeric type of port and run boundary mode analysis first to find boundary mode near 
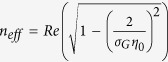
. This *n*_*eff*_ is essentially the real part of effectively refractive index of air-graphene-air surface waveguide, indicated by Eq. 16, so it is necessary to leave sufficiently long waveguide before and after the interested structures to do boundary mode analysis. The top and bottom boundaries are set to be scattering boundaries. In the simulation, graphene is also assumed to have finite thickness (1 *nm*), which is not the actual physical thickness of graphene (about 0.33 *nm*). It is shown that as long as the mesh gird is sufficiently small, the difference between results obtained for 1 *nm* and 0.33 *nm* is negligible^41^,^44^,^54^.

### Analysis of transmission in terms of wave impedance

From Fig. 5(a), it can be concluded that 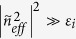 holds for every dielectric material in the model. Thus Eq. 15 can be approximated as


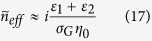


In the infrared region of our interest, the optical excitation of graphene is mostly contributed by intraband transitions^38^,^55^,^56^. Combining Eq. 1 and Eq. 17 leads to 
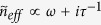
, so 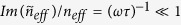 for typical values *ω* = 3 × 10^14^*rad*/*s* and *τ* = 6 × 10^−13^ *s*. This indicates that the propagation length of SPPs in graphene is much longer than their wavelength, making it reasonable to use wave impedance to analyze the reflection and transmission of SPPs in the structure. In addition, Fig. 4(a) shows that the transmitted wavelength does not vary with carrier mobility. So wave impedance analysis can be used to understand reflection and transmission of propagrating SPPs in the structure. We define wave impedance for transverse waves, whose oscillations occuring perpendicular to the propagation direction, as *Z* = *E*_*T*_/*H*_*T*_ at a particular point. The longitudinal components of electric field get transmitted according to the continuity of displacement field. The propagation of wave impedance from *Z*(*l*) to *Z*(0) in one medium is given by^57^


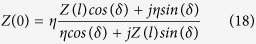


where *η* is the intrinsic impedance of the medium, defined as 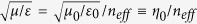. Equation 18 implies that only the phase shift *δ* = *k*_0_*n*_*eff*_*l* ∈ [0, 2*π*) matters in the analysis of impedance propagation. Two particular cases are when the propagation distances (optical lengths) equal to half and quarter of the wavelength


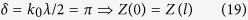



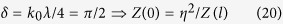


For a Bragg reflector made of alternating layers of high (*H*) and low (*L*) effective refractive indices, taking Reflector II shown in Fig. 5(b) as an example, Eq. 21 holds for a certain wavelength *λ*


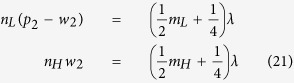


where *m*_*L*_ and *m*_*H*_ are certain integers. So both geometry parameters and effective refractive index can shift the stopband, as discussed in Fig. 3. Based on Eq. 20 and the fact that wave impedance is continuous across the interface, we have the expression for the wave impedance at the right boundary of the bridge


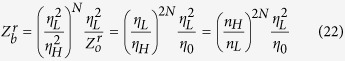


where *N* is the number of periods in Reflector II. So 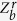 increases very fast as N becomes large (*n*_*H*_ > *n*_*L*_). Similarly, for Reflector I


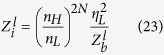


When the optical length


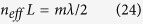


from Eq. 19, 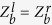. Substitution of Eq. 22 into Eq. 23 gives 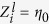, which in turn leads to the vanishing of reflection coefficient for transverse components 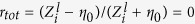. Since the materials before and after the structure are both assumbed to be air (intrinsic impedances are both set to be *η*_0_), continuity of displacement field ganrantees the perfect transmission of longitudinal components. So the total transmission is 1 for the particular wavelength *λ*. Note Eq. 24 is the same as Eq. 4, so wave impedance analysis verifies that the simple picture of taking two Bragg reflectors as highly reflecting mirrors gives the right estimation of the wavelength of the transmission peak.

## Additional Information

**How to cite this article**: Wang, X. *et al*. A graphene-based Fabry-Pérot spectrometer in mid-infrared region.*Sci. Rep.* **6**, 32616; doi: 10.1038/srep32616 (2016).
